# Fiendish Enterprise

**Published:** 1888-08

**Authors:** 


### FIENDISH ENTERPRISE.

"Scotch Oats Essence" is not only a swindle in terms—containing not a particle of its pretended constituent—but is also a deliberately, devised agent (says the *Pharmaceutical Era*) "for making men and women victims of the opium habit, to which the incarnate fiend who planned the scheme should thereafter become the sole purveyor. The preparation designed to produce this disastrous effect is simply advertised as a nerve recuperator, under a name, Scotch Oats Essence, which would seem to guarantee its innocent character. Of course it will find its way to exactly the class of patients predisposed to morphiomania, and if used, according to the directions, in increasing quantities until its full effect shall be felt, will not fail shortly to fix on many a victim the chains of a slavery from which few have ever been able to free themselves.

"The dangerous character of the preparation was pointed out by Dr. R. G. Eccles, who published in the *Druggists' Circular* for April an analysis showing that it contained one-third to one-half a grain of morphine in each fluid ounce. We heartily concur in the expressions of strong condemnation which this revelation calls from the editor of that journal, and commend to legislative committees of pharmaceutical organizations throughout the country the formulating of laws which shall severely punish a crime so utterly diabolical."

Dr. George W. Winterburn says in *Home Knowledge*: "There is a tribe of infant murderers, Godfrey's Cordial, paragoric, and Mrs. Winslow's Soothing Syrup. The mixtures are used in the vast majority of cases because they are supposed to be harmless. Many do not know that paragoric contains opium. For murderous efficiency Mrs. Winslow's bears the palm. Many a little sufferer whose demise is chronicled in the Board of Health as from meningitis, marasmus, dysentery, or fever, was killed by the slow undermining of the constitution by one of these opiated preparations. Opiated mixtures ought not to be sold except on a physician's prescription, and the druggists should be prevented by law from renewing prescriptions containing opium in any form, while doctors are recommended to abandon the prescription of opium as far as possible." —*The Sanitary Era.*

Are not our Health Boards invested with authority to prohibit the sale and use of these nostrums so clearly dangerous to health ? If not, such power should be conferred upon them, that these murderous compounds may be driven from the market and these proprietors duly punished. It is vastly more important to avert an evil, than to publish annual statistics of its life destroying ravages.

